# Distinct neural bases of disruptive behavior and autism symptom severity in boys with autism spectrum disorder

**DOI:** 10.1186/s11689-017-9183-z

**Published:** 2017-01-17

**Authors:** Y. J. Daniel Yang, Denis G. Sukhodolsky, Jiedi Lei, Eran Dayan, Kevin A. Pelphrey, Pamela Ventola

**Affiliations:** 1 Autism and Neurodevelopmental Disorders Institute, The George Washington University and Children’s National Health System, 2300 I St NW, Washington, DC 20052 USA; 2 Child Study Center, Yale University School of Medicine, New Haven, CT 06519 USA; 3 Division of Psychology and Language Sciences, University College London, London, WC1H 0AP UK; 4 Department of Radiology and Biomedical Research Imaging Center, The University of North Carolina at Chapel Hill, Chapel Hill, NC 27599 USA

**Keywords:** Autism spectrum disorder, Comorbidity, Neuroimaging, Social perception, Disruptive behavior, Oppositional defiant disorder, Anxiety disorders, ADHD, Default mode network

## Abstract

**Background:**

Disruptive behavior in autism spectrum disorder (ASD) is an important clinical problem, but its neural basis remains poorly understood. The current research aims to better understand the neural underpinnings of disruptive behavior in ASD, while addressing whether the neural basis is shared with or separable from that of core ASD symptoms.

**Methods:**

Participants consisted of 48 male children and adolescents: 31 ASD (7 had high disruptive behavior) and 17 typically developing (TD) controls, well-matched on sex, age, and IQ. For ASD participants, autism symptom severity, disruptive behavior, anxiety symptoms, and ADHD symptoms were measured. All participants were scanned while viewing biological motion (BIO) and scrambled motion (SCR). Two fMRI contrasts were analyzed: social perception (BIO > SCR) and Default Mode Network (DMN) deactivation (fixation > BIO). Age and IQ were included as covariates of no interest in all analyses.

**Results:**

First, the between-group analyses on BIO > SCR showed that ASD is characterized by hypoactivation in the social perception circuitry, and ASD with high or low disruptive behavior exhibited similar patterns of hypoactivation. Second, the between-group analyses on fixation > BIO showed that ASD with high disruptive behavior exhibited more restricted and less DMN deactivation, when compared to ASD with low disruptive behavior or TD. Third, the within-ASD analyses showed that (a) autism symptom severity (but not disruptive behavior) was uniquely associated with less activation in the social perception regions including the posterior superior temporal sulcus and inferior frontal gyrus; (b) disruptive behavior (but not autism symptom severity) was uniquely associated with less DMN deactivation in the medial prefrontal cortex (MPFC) and lateral parietal cortex; and (c) anxiety symptoms mediated the link between disruptive behavior and less DMN deactivation in both anterior cingulate cortex (ACC) and MPFC, while ADHD symptoms mediated the link primarily in ACC.

**Conclusions:**

In boys with ASD, disruptive behavior has a neural basis in reduced DMN deactivation, which is distinct and separable from that of core ASD symptoms, with the latter characterized by hypoactivation in the social perception circuitry. These differential neurobiological markers may potentially serve as neural targets or predictors for interventions when treating disruptive behavior vs. core symptoms in ASD.

**Electronic supplementary material:**

The online version of this article (doi:10.1186/s11689-017-9183-z) contains supplementary material, which is available to authorized users.

## Background

Recent development in the field of autism spectrum disorder (ASD) has been making strides in revealing the neural basis of its core symptoms, namely social communication deficits and restrictive and repetitive behavior [1]. For example, neuroimaging studies consistently show that the posterior superior temporal sulcus (pSTS), fusiform gyrus (FFG), and inferior frontal gyrus (IFG) are key regions for social information processing [2] and individuals with ASD relative to typically developing (TD) controls exhibited hypoactivation in these regions [3, 4]. However, ASD often co-occurs with other psychiatric disorders [5, 6], such as anxiety disorder [7], attention-deficit/hyperactivity disorder (ADHD) [8], and oppositional defiant disorder (ODD) [9]. So far, knowledge regarding the neural basis of the comorbidity in ASD is relatively lacking in the literature, and little is known about whether or not the co-occurring disorders in ASD and core ASD symptoms share the same neural basis or not. Understanding the neural basis of the comorbidity in ASD and defining the boundaries between ASD and its comorbid psychiatric disorders may identify targets for specific intervention in subgroups of ASD that could improve quality of life, reduce impairment, and increase treatment effectiveness for ASD. In the current research, we focus on disruptive behavior in ASD and delineate its neural underpinning. We also address the question of whether the neural basis of disruptive behavior in ASD is shared with or separable from that of core symptoms in ASD.

Disruptive behavior in children with ASD is relatively common, from about one fourth to one third of children with ASD displaying disruptive behavior [6, 10], including angry outburst, irritability, as well as oppositional, noncompliant, and aggressive behaviors [11]. On the one hand, disruptive behavior in children with ASD may allow them to escape demands (e.g., escaping from learning), retain access to items, and avoid sensory stimuli (e.g., noises in the environment) [10, 12, 13]. Thus, some could argue that for children with ASD, disruptive behavior may serve the purpose to gain access to restricted and repetitive interests or to escape from uncomfortable social and sensory demands and should be conceptualized as core ASD symptoms. On the other hand, others could argue that disruptive behavior, characterized by a long-lasting, context-independent pattern of angry/irritability, argumentative/defiant behavior, or vindictiveness, should be viewed as a comorbidity of and different from core ASD symptoms [10]. In this study, we refer disruptive behavior to the latter definition and we assessed it with a chronic behavioral pattern independent of the functional properties or setting in which disruptive behavior occurs, while we assumed that it is a comorbidity that could be distinguished from core ASD symptoms [14].

To evaluate the neural basis of core ASD symptoms, we used a biological motion fMRI task [3]. Although being relatively impoverished stimuli, point-light displays contain sufficient information to identify the kind of motion being produced (e.g., walking, dancing, reaching), as well as the identity of the agent [15]. Our prior neuroimaging work identified dysfunction in the biological motion processing system as reflecting key neural signatures of ASD in affected children in terms of hypoactivation in the ventrolateral prefrontal cortex, amygdala, IFG, pSTS, and FFG [3]. Other fMRI studies also showed ASD-related abnormalities in the neural pathways of processing biological motion [16, 17], particularly in the pSTS region [18–20]. In the scanner, our study participants viewed stimuli depicting point light displays of coherent biological (BIO) or scrambled biological (SCR) motion, created from motion capture data (i.e., videos created by placing lights on the major joints of a person and filming them moving in the dark) [3, 21].

In contrast, to evaluate the neural basis of disruptive behavior, we used the same fMRI task but relied on a novel fMRI contrast: fixation > BIO, which provides a window into deactivation of the default mode network (DMN). DMN deactivation is related to self-regulation [22–24] and represents an important neural process where self-related neural activity is suppressed during focused attention on the external environment [25]. The core DMN nodes include three interconnected regions [26–28]: medial prefrontal cortex (MPFC), posterior cingulate cortex/precuneus (PCC/PC), and the lateral parietal cortex (LPC) [27–30]. The MPFC is found to be associated with self-appraisal [31, 32] and self-referential thoughts [33]; the PCC/PC has been linked to arousal and conscious perception of interoceptive stimulation [34]; and the LPC has been reported to be related to recollection of episodic memories and retrieval of spatial context memory [35–37]. Research suggests their roles in processing internally generated self-referential thoughts and mind-wandering in healthy individuals [38]. An important property of the DMN is that the network has been shown to deactivate during cognitively demanding tasks that involve an external target in healthy individuals [25, 27, 39], suggesting that people may engage in down-regulation of self-referential thoughts when processing the external task, thus reducing interference [40]. For this reason, we chose the contrast of fixation > BIO to tap into DMN deactivation because BIO is relatively more cognitively demanding than fixation and involves an external target. Accurate perception of biological motion requires individuals to first track motion timings, then integrate perceived timings into a coherent kinematic framework for higher-order processing. Consistent with this notion, previous research showed that DMN deactivation was necessary for healthy adults in order to process biological motion [41].

On the other hand, failure to deactivate DMN when engaged in tasks has been reported in several psychopathologies, such as depression, where pathological rumination of negative self-related thoughts during task has been linked to poor self-regulation [25]. Several recent studies have also suggested that DMN alternation is implicated in children and adolescents with disruptive behavior [42–45]. However, to the best of our knowledge, no study has examined the link between DMN activity and disruptive behavior in ASD. Using the biological motion task and the novel contrast of DMN deactivation (fixation > BIO), the current study further tested this link in children with ASD.

Importantly, the contrast of fixation > BIO should be interpreted as DMN deactivation in only relative but not absolute terms. Here, the potential DMN activation during fixation is treated as a comparison point, and a positive (or negative) value of this contrast in the DMN may indicate that there is less (or more) activation in the DMN during BIO than during fixation periods, arguably reflecting down regulation of DMN activation during BIO vs. during fixations. In this research, we call it the contrast of DMN deactivation (fixation > BIO) and emphasize that it should not be interpreted as DMN deactivation during BIO alone. The contrast can only be interpreted in terms of differential activation and cannot be used to reveal the absolute levels of DMN activation within BIO or fixation periods, respectively.

In sum, this study examined the neural basis of disruptive behavior in ASD and investigated whether it is shared with or separable from that of core ASD symptoms. The two contrasts in the biological motion task, namely, social perception (BIO > SCR) and DMN deactivation (fixation > BIO), afford the opportunity to compare these two neural bases within the same sample of children with ASD. We hypothesize that there would be distinct and separable neural bases of disruptive behavior and autism symptom severity in ASD, in which (a) autism symptom severity would be associated with less activation in the social perception circuitry [2] and (b) disruptive behavior would be associated with less DMN deactivation. We also explored co-occurring anxiety and ADHD symptoms [6] as potential mediators of the neural basis of disruptive behavior.

## Methods

### Participants

Study participants included 48 children and adolescents (all males) between 4 and 18 years of age. They consisted of 31 boys with autism spectrum disorder (4.54–18.43 years) and 17 TD boys (5.07–16.68 years). Nine participants with ASD and 8 TD participants also participated in a prior imaging study [3] that investigated the neural basis of ASD. All participants received the same fMRI imaging paradigm in the same scanner. IQ was measured using the Differential Ability Scales-Second Edition (DAS-II) [46]. DAS-II was used for this project because it covers the age range of children included in the study. DAS-II is also commonly used in studies of children with ASD, as it requires less language than other cognitive measures [47–50]. All participants were high-functioning (IQ > 70); the ranges of Full-Scale IQ (FSIQ) were 74–131 for ASD and 78–127 for TD. The ASD and TD groups were well-matched on age, IQ, and head motion during fMRI scan (see Table 1).

**Table 1:** Participants demographics and group matching

	TD (*n* = 17)		ASD (*n* = 31)		TD vs. ASD	
Variable	Mean (SD)	Range	Mean (SD)	Range	*t*_(46)_	*p*
Age (years)	10.92 (2.85)	5.07–16.68	10.86 (3.63)	4.54–18.43	0.06	0.95
IQ	104.12 (12.87)	78–127	98.10 (16.32)	74–131	1.31	0.20
Verbal IQ	104.53 (10.44)	87–120	101.52 (17.18)	72–141	0.66	0.51
Non-verbal IQ	103.65 (14.27)	74–126	96.65 (16.80)	73–138	1.45	0.15
Head motion (*M* absolute, mm)	0.46 (0.54)	0.08–2.08	0.48 (0.49)	0.09–1.84	−0.14	0.89
Head motion (*M* relative, mm)	0.10 (0.07)	0.03–0.24	0.14 (0.12)	0.03–0.46	−1.34	0.19

*M* mean

All participants with ASD met DSM-5 [51] diagnostic criteria for ASD as determined by expert clinical judgment. This judgment was supported by the results of gold-standard diagnostic instruments, Autism Diagnostic Interview-Revised (ADI-R) [52] and Autism Diagnostic Observation Schedule (ADOS) [53–56], administered by research-reliable and licensed clinical psychologists. The complete characterization of the ASD group is reported in Table 2.

**Table 2:** ASD group characteristics

Variable	All (*n* = 31)	Low ODD (*n* = 24)	High ODD (*n* = 7)	Low vs. high		
				*t*	*df*	*p*
ADI-R	*n* = 30	*n* = 24	*n* = 6			
Social	22.03 (3.99)	22.08 (3.91)	21.83 (4.67)	0.14	28	0.89
Verbal communication	17.73 (4.62)	17.96 (4.61)	16.83 (4.96)	0.53	28	0.60
Repetitive behaviors	6.23 (2.81)	6.38 (2.68)	5.67 (3.50)	0.55	28	0.59
ADOS module 2	*n* = 1	*n* = 1	---			
SA domain	11.00 (---)	11.00 (---)	---	---	---	---
RRB domain	5.00 (---)	5.00 (---)	---	---	---	---
Total	16.00 (---)	16.00 (---)	---	---	---	---
ADOS module 3	*n* = 29	*n* = 22	*n* = 7			
SA domain	9.76 (3.65)	9.68 (3.39)	10.00 (4.69)	−0.20	27	0.85
RRB domain	2.52 (1.70)	2.64 (1.71)	2.14 (1.77)	0.66	27	0.51
Total	12.28 (4.33)	12.32 (3.95)	12.14 (5.73)	0.09	27	0.93
ADOS module 4	*n* = 1	*n* = 1	---			
SA domain	11.00 (---)	11.00 (---)	---	---	---	---
RRB domain	1.00 (---)	1.00 (---)	---	---	---	---
Total	12.00 (---)	12.00 (---)	---	---	---	---
ADOS Calibrated Severity Score	7.19 (1.85)	7.29 (1.68)	6.86 (2.48)	0.54	29	0.59
SRS-parent total raw score	97.87 (30.13)	93.33 (30.70)	113.43 (23.73)	−1.59	29	0.12
Disruptive behavior	9.32 (5.17)	7.17 (3.51)	16.71 (2.06)	−6.81****	29	< 0.0001
Anxiety symptoms	7.26 (4.97)	6.21 (4.08)	10.86 (6.34)	−2.34*	29	0.03
ADHD symptoms	25.25 (8.10)	23.38 (6.24)	31.66 (10.80)	−2.60*	29	0.02
Head motion (*M* absolute, mm)	0.48 (0.49)	0.47 (0.45)	0.52 (0.66)	−0.26	29	0.80
Head motion (*M* relative, mm)	0.14 (0.12)	0.14 (0.11)	0.14 (0.15)	−0.02	29	0.98

The numbers are mean (SD)

*ADI-R* Autism Diagnostic Interview-Revised, *ADOS* Autism Diagnostic Observation Schedule, *SRS* Social Responsiveness Scale, *ADHD* attention-deficit/hyperactivity disorder, *ODD* oppositional defiant disorder, *SA* social affect, *RRB* restricted and repetitive behaviors, *M* mean

*p < 0.05; ****p < 0.0001

To rule out possible developmental delays, psychiatric disorders, and the broad autism phenotype (BAP) [57, 58] in the TD participants, we used the following exclusion criteria based on the criteria used in previous research in our lab [3]: (a) diagnosed or suspected ASD, or other psychiatric or neurological disorder; (b) first- or second-degree relative with diagnosed or suspected ASD; (c) an individualized education program for special education services, including speech/language therapy, occupational therapy, and/or social skills intervention; or (d) Social Responsiveness Scale (SRS)-parent total *t* score ≥76 (severe range). In our TD sample, the SRS total *t* scores had *M* = 45.64, *SD* = 6.71, and range = 37–60, which were far below the exclusion threshold and generally within the normal range (*t* score ≤59).

Exclusion criteria for all participants included a history of serious head injury or loss of consciousness. All participants passed MRI safety screening, including being free of any metal implants and evidence of claustrophobia. Written informed consent was obtained from each participant’s parent(s), and assent was obtained from each participant. The Human Investigations Committee at Yale University approved this study.

### Behavioral clinical measures

#### Autism symptom severity

The severity of ASD symptoms was measured using the parent-reported Social Responsiveness Scale (SRS) total raw scores [59, 60]. The scale has 65 items and assesses social awareness, social information processing, capacity for reciprocal social communication, social motivation, and autistic mannerisms. Rather than using a "yes or no" decision about the presence of symptom, the SRS uses a 4-point scale from 1 (“*not true*”) to 4 (“*almost always true*”), and the total raw scores across 65 items provide a fine-grained, continuous measure of the child’s symptom severity, consistent with the notion that autism is best conceptualized as a spectrum condition. In contrast, ADI-R and ADOS scores are primarily for ASD diagnosis and provide a more limited range of scores. For this reason, we chose the SRS total raw score as a measure of autism symptom severity because it provides a greater range of scores across multiple domains of ASD symptoms.

#### Disruptive behavior and potential mediators

Disruptive behavior was measured with the ODD subscale of the Child Symptom Inventory-4 (CSI-4) [61] for participants aged 5 to 12 years and the Adolescent Symptom Inventory-4 (ASI-4R) [62] for those aged 12 to 18 years. The CSI-4 and ASI-4R are parent-reported behavior rating scales whose items correspond to the symptoms of disorders defined by the DSM-IV. The ODD subscale in the CSI-4 or ASI-4R includes eight items. Example items are “loses temper,” “argues with adults,” and “takes anger out on others or tries to get even.” On each item, parent rated how well it describes the child’s overall behavior on a 4-point frequency scale from 0 (“*never*”) to 3 (“*very often*”). The ODD scale has been used in children with ASD and cut-off scores (>13 in CSI-4 and >12 in ASI-4R) have been used to identify clinically significant symptoms of ODD [9, 61–63].

In addition, to test candidate mediators of a possible link between disruptive behavior and its neural basis in ASD, we included measures of anxiety and ADHD symptoms, respectively. First, anxiety symptoms were measured via the Generalized Anxiety Disorder (GAD) subscale in CSI-4 and ASI-4R [61, 62], which includes 8 items (*M* = 7.26, *SD* = 4.97) using the same 4-point scale. Example GAD items are “has difficulty controlling worries”, and “is extremely tense or unable to relax”. Second, ADHD symptoms were measured via the combined ADHD subscale (combining both inattentive and hyperactivity/impulsivity dimensions) in CSI-4 and ASI-4R [61, 62], which includes 18 items (*M* = 25.25, *SD* = 8.10) using the same 4-point scale. Example ADHD items are “fails to give close attention to details or makes careless mistakes” and “fidgets with hands or feet or squirms in seat.” As expected, in ASD participants, ODD symptoms were significantly correlated with anxiety symptoms, *r* = 0.45, *p* = 0.01, and with ADHD symptoms, *r* = 0.50, *p* < 0.01, supporting that both anxiety and ADHD symptoms may serve as potential mediators.

For our analyses, the ASD sample was further divided into those with low (*n* = 24) vs. high (*n* = 7) disruptive behavior based on the cutoffs in the ODD subscale (>13 in CSI-4 and >12 in ASI-4R) [61, 62]. As seen in Table 2, the two ASD subgroups were statistically comparable on autism symptom severity, *p* = 0.12, but significantly different on disruptive behavior, anxiety symptoms, and ADHD symptoms, *p*s < 0.05.

### fMRI experimental design

**Fig. 1:**
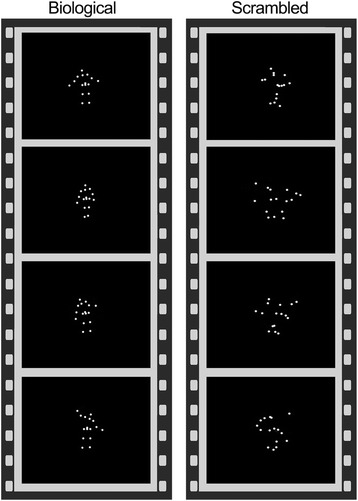
Example of fMRI stimuli used in the current study

Participants were scanned while viewing coherent and scrambled point-light displays of biological motion created from motion capture data. The coherent biological (BIO) motion displays featured an adult male actor performing movements and contain 16 points corresponding to major joints. The scrambled (SCR) motion animations were created by randomly plotting the trajectories of all the 16 points from the coherent biological motion displays on a black background (see Fig. 1 for an example). Thus, the coherent and scrambled displays contained the same local motion information, but only the coherent displays contained the configuration of a person [15]. Stimuli were presented using E-Prime 2.0 software (Psychological Software Tools, Pittsburgh, PA, USA) during the scan. Six coherent biological motion clips (BIO) and six scrambled motion clips (SCR) were presented once each in an alternating block design (time per block, ~24 s). The experiment began with a 20-s fixation period and ended with a 16-s fixation period. The total duration was about 328 s. The movies were presented without audio. The participants were asked to watch the videos and reminded to remain still and alert.

### Imaging acquisition

Scanning was performed on a Siemens MAGNETOM Trio, A Tim System 3 T scanner at the Yale Magnetic Resonance Research Center. For each participant, a structural MRI image was acquired with a 32-channel head coil, a T1-weighted MPRAGE sequence, and the following parameters: 160 sagittal slices; TR = 1900 ms; TE = 2.96 ms; flip angle = 9°; slice thickness = 1.00 mm; voxel size = 1 × 1 × 1 mm^3^; matrix = 256 × 256; and field of view = 256 × 256 mm^2^. Afterwards, BOLD T2*-weighted functional MRI images for the biological motion task were acquired using the following parameters: 164 volumes; TR = 2000 ms; TE = 25 ms; flip angle = 60°; slice thickness = 4.00 mm; voxel size = 3.44 × 3.44 × 4.00 mm^3^; matrix = 64 × 64; field of view = 220 × 220 mm^2^; number of slices per volume = 34; and interleaved acquisition.

### Imaging processing

The T1-weighted MPRAGE structural scan was segmented by SPM12 into gray matter, white matter (WM), and cerebrospinal fluid (CSF) images. This method has been shown to be highly accurate and has little bias when compared to manual measurement [64].

The fMRI data were processed using FSL [65] v5.0.8 and the participant-level preprocessing steps followed a standardized processing stream described in the paper of ICA-AROMA (ICA-based strategy for Automatic Removal of Motion Artifacts) [66] and consisted of the following: (1) motion correction using MCFLIRT, (2) interleaved slice timing correction, (3) BET brain extraction, (4) grand mean intensity normalization for the whole 4D data set, (5) spatial smoothing with 5 mm FWHM, (6) data denoising with ICA-AROMA [66], which uses a robust set of theoretically motivated temporal and spatial features to remove motion-related spurious noise, (7) nuisance regression using time-series for WM and CSF signal to remove residual, physiological noise, and finally (8) high-pass temporal filtering (100 s). The first 4 s were discarded to establish T1 equilibrium. Registration of the fMRI data was performed using both the subject’s structural scan and then the Montreal Neurological Institute (MNI152) standard brain. Preprocessed data were then pre-whitened using FSL’s FILM to remove time series autocorrelation.

To model the BIO and SCR conditions, the timing of the corresponding blocks was convolved with the default gamma function (phase = 0 s, standard deviation = 3 s, mean lag = 6 s) with temporal derivatives. Fixation was modeled as an implicit baseline. The two participant-level contrasts of interest were BIO > SCR and fixation > BIO, which served as inputs for the subsequent mass univariate voxel-wise group-level GLM (General Linear Model) analyses. Because there was a wide age range in the participants, age was included as a covariate of no interest. To control for the possibility that IQ may alter the difficulty of processing biological motion [67], IQ was also included as a covariate of no interest.

### Group-level GLM analyses

The group-level GLM analyses were conducted using mixed-effects modeling by FSL’s FLAME (FMRIB’s Local Analysis of Mixed Effects) 1 + 2 algorithm to ensure that the results are generalizable to the population and are the most accurate estimate of activation. Because the research is pioneering and the first of its kind in ASD and it is desirable not to miss possible true effects [68], while there were only 7 participants in the subgroup of ASD with high ODD, the analyses were based on a relatively lenient cluster-defining threshold (CDT) of *Z* > 1.96 and corrected for multiple comparisons with a cluster-level significance threshold of *p* < 0.05. Information about the surviving clusters was reported, including the anatomical regions covered by the clusters based on the Automated Anatomical Labeling v2 (AAL2) atlas [69], the coordinates of the peak voxels within each of the anatomical regions, and the Z-statistics associated with the peak voxels. Voxel size = 2 × 2 × 2 mm^3^. Age and IQ were controlled for as covariates of no interest in all analyses. Continuous variables were mean-centered before included in the group-level GLM analyses.

To understand the neural basis of disruptive behavior in children with ASD, we first analyzed between-group differences on the fMRI contrast of social perception (BIO > SCR) and then on the contrast of DMN deactivation (fixation > BIO). The between-group analyses were based on the following group-level GLM equations (1) and (2), where **y** is the voxel-level activation, *β*’s are the parameter estimates, and *ε* is the residual. These GLM equations were estimated on the two contrasts respectively, namely, social perception (BIO > SCR) and DMN deactivation (fixation > BIO).

GLM (1): **y =** *β*_1_ × (TD = 1; otherwise = 0) + *β*_2_ × (ASD_All_ = 1; otherwise = 0) +*β*_3_ × age + *β*_4_ × IQ + *ε*

GLM (2): **y =** *β*_1_ × (TD = 1; otherwise = 0) + *β*_2_ × (ASD_Low-ODD_ = 1; otherwise = 0) + *β*_3_ × (ASD_High-ODD_ = 1; otherwise = 0) + *β*_4_ × age + *β*_5_ × IQ + *ε*

Next, within the ASD sample, we analyzed the neural correlates of disruptive behavior and autism symptom severity, respectively. However, ODD total scores (tapping disruptive behavior) and SRS total raw scores (tapping autism symptom severity) were marginally correlated, *r* = 0.32, *p* = 0.08, suggesting that the two measures were differentiable but also partly overlapped. To be comprehensive, we examined the effects of ODD total scores and SRS total raw scores first separately and then simultaneously by covarying out the effects of the other dimension. The within-ASD analyses were based on the following GLM equations (3), (4), and (5). These GLM equations were estimated on the two contrasts respectively, namely, the contrast of social perception (BIO > SCR) and that of DMN deactivation (fixation > BIO).

GLM (3): **y =** *β*_0_ + *β*_1_ × age + *β*_2_ × IQ + *β*_3_ × SRS_total-raw-scores_ + *ε*

GLM (4): **y =** *β*_0_ + *β*_1_ × age + *β*_2_ × IQ + *β*_3_ × ODD_total-scores_ + *ε*

GLM (5): **y =** *β*_0_ + *β*_1_ × age + *β*_2_ × IQ + *β*_3_ × SRS_total-raw-scores_ + *β*_4_ × ODD_total-scores_ + *ε*

Finally, to explore how anxiety and ADHD symptoms, respectively, mediated the neural correlates of disruptive behavior, we relied on the well-established procedure described in the literature [70] and the following GLM equations (6) and (7). These GLM equations were estimated on the contrast of DMN deactivation (fixation > BIO).

GLM (6): **y =** *β*_0_ + *β*_1_ × age + *β*_2_ × IQ + *β*_3_ × ODD_total-scores_ + *β*_4_ × Anxiety_symptoms_ + *ε*

GLM (7): **y =** *β*_0_ + *β*_1_ × age + *β*_2_ × IQ + *β*_3_ × ODD_total-scores_ + *β*_4_ × ADHD_symptoms_ + *ε*

### Power considerations

Our fMRI power analyses involve calculations for the number of participants needed to detect the group difference between TD and ASD on this contrast of BIO > SCR. Our prior study of biological motion perception [3] showed large group differences (Cohen’s *d* ≥ 1.5) in 25 children with ASD relative to 17 TD controls in the right pSTS, right amygdala, right FFG, right IFG, and ventromedial prefrontal cortex. According to G*Power [71], at *α* = 0.05, two-sided, with 17 TD and 7–31 ASD participants, we would have 89.1–99.8% power to detect the between-group difference. This ensures that this study is sufficiently powered to test group differences on the contrast of BIO > SCR. In contrast, for the novel contrast of fixation > BIO, there was no prior study available to calculate the required sample size, and we tested its effects in this study for the first time, although our confidence was boosted because several recent studies have consistently suggested that DMN alternation is implicated in children and adolescents with disruptive behavior [42–45].

## Results

### Between-group differences on the contrast of social perception (BIO > SCR)

**Fig. 2:**
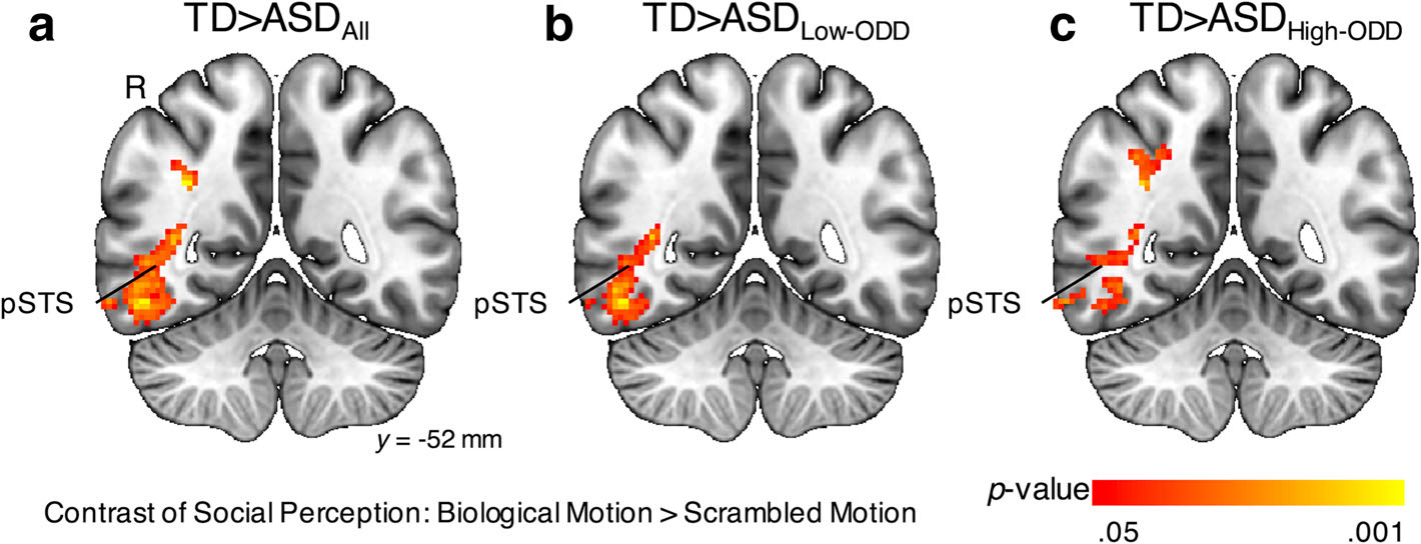
Between-group results on the contrast of social perception (BIO > SCR). BIO, biological motion; SCR, scrambled motion; pSTS, posterior superior temporal sulcus; *R*, right hemisphere; TD, typically developing; ASD, autism spectrum disorder; ODD, oppositional defiant disorder. **a** TD > ASD_All_. **b** TD > ASD_Low-ODD_. **c** TD > ASD_High-ODD_

To limit the inferential space to regions showing group main effects, we masked the analysis by a combined, inclusive (TD∪ASD; the union of the two sets) mask consisting of regions that showed main effects for BIO > SCR within either group (see Additional file 1). Here, we found that boys with ASD relative to TD controls have reliably weaker activation in the pSTS and FFG regions on the right hemisphere (Fig. 2a; Table 3). Interestingly, the hypoactivation in these two regions was largely unaffected by the presence of disruptive behavior, as both ASD with low disruptive behavior and ASD with high disruptive behavior showed similar hypoactivation in these regions (Fig. 2b, c; Table 3), and direct comparison between ASD with high vs. low disruptive behavior on this contrast revealed no regions of significant difference. In brief, the between-group results on the contrast of BIO > SCR showed that ASD is characterized by hypoactivation in specific social perception regions, while ASD with high or low disruptive behavior exhibited similar hypoactivation in these regions.

**Table 3:** Peaks of regions in which the contrast of social perception (BIO > SCR) exhibited TD > ASD group differences

		TD > ASD_All_				TD > ASD_Low-ODD_				TD > ASD_High-ODD_			
Anatomical regions		*x*	*y*	*z*	*Z*	*x*	*y*	*z*	*Z*	*x*	*y*	*z*	*Z*
Angular gyrus	R	34	−60	42	3.09					34	−58	42	4.13
Fusiform gyrus	R	34	−44	−4	2.72	42	−54	−16	2.56	34	−44	−4	2.82
Hippocampus	R	36	−34	−4	3.11								
Inferior occipital gyrus	R	52	−78	−4	3.60	46	−86	−2	3.61				
Middle occipital gyrus	R	38	−92	8	4.38	44	−88	6	4.35	34	−62	38	2.87
Inferior parietal gyrus	R	38	−54	40	2.71					36	−54	42	3.38
Supramarginal gyrus	R									38	−42	42	3.06
Inferior temporal gyrus	R	58	−64	−14	3.93	58	−64	−14	3.68	42	−58	−8	3.15
Middle temporal gyrus^a^	R	56	−72	0	3.95	56	−72	0	3.97	44	−68	16	3.37

Coordinates are in MNI152 mm space. Results were thresholded at *Z* > 1.96 (*p* < 0.05) and corrected for multiple comparisons at the cluster level (*p* < 0.05)

*R* right, *BIO* biological motion, *SCR* scrambled motion, *ODD* oppositional defiant disorder

^a^The hypoactivation in the right posterior superior temporal sulcus (pSTS) was detected in all these three comparisons (see Fig. 2). Because the right pSTS is not one of the pre-defined anatomical regions of the AAL2 atlas, it was not listed here. In our results, the hypoactivation in the right pSTS was primarily in the anatomical region of the right middle temporal gyrus

### Between-group differences on the contrast of DMN deactivation (fixation > BIO)

**Fig. 3:**
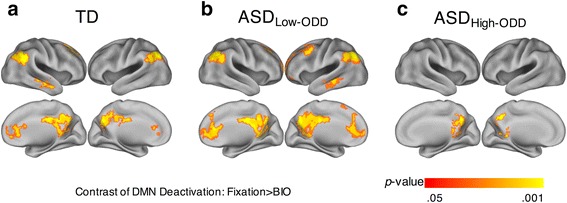
Group-based results on the contrast of DMN deactivation (fixation > BIO). DMN, default mode network; BIO, biological motion. **a** TD. **b** ASD_Low-ODD_. **c** ASD_High-ODD_

To ensure that the results can be readily interpreted as DMN deactivation, the between-group analysis on the contrast of DMN deactivation (fixation > BIO) was masked by a DMN mask [26]. The DMN mask was well established in the literature with 1000 healthy young adults and includes several key DMN regions, including (but not limited to) the ventral medial prefrontal cortex, the dorsal medial prefrontal cortex, the posterior cingulate cortex, and adjacent precuneus plus the lateral parietal cortex [26, 27]. Furthermore, to limit the inferential space to regions showing group main effects, we masked the analysis by a combined, inclusive (TD∪ASD; the union of the two sets) mask consisting of regions that showed main effects for fixation > BIO within the DMN within either group (see Additional file 1). Here, our analyses revealed that TD and ASD had comparable deactivations across multiple DMN regions (see Additional file 2), while there was no region showing significant group differences between TD and ASD. Direct comparison between TD and ASD with low disruptive behavior on this contrast also revealed no regions of significant difference. However, compared to TD (Fig. 3a) and ASD with low disruptive behavior (Fig. 3b), respectively, ASD with high disruptive behavior (Fig. 3c) exhibited more restricted regions of DMN deactivation. This is supported by direct comparison between ASD with high vs. low disruptive behavior, which showed that ASD with low (vs. high) disruptive behavior had significantly greater deactivation in several DMN regions such as the medial prefrontal cortex and the inferior parietal gyrus (Table 4). Furthermore, at a more liberal threshold (*Z* > 1.96, *p* < 0.05, uncorrected; minimum clusters = 17 voxels), direct comparison between TD and ASD with high disruptive behavior support that TD had significantly greater deactivation in several DMN regions including the right angular gyrus, the left supramarginal gyrus, the bilateral anterior cingulate gyri. In brief, the between-group results on the contrast of DMN deactivation showed that ASD with high disruptive behavior exhibited more restricted and less DMN deactivation, when compared to ASD with low disruptive behavior or TD.

**Table 4:** Peaks of DMN regions in which the contrast of DMN deactivation (fixation > BIO) exhibited ASD_Low-ODD_ > ASD_High-ODD_ group differences

Anatomical regions		*x*	*y*	*z*	*Z*
Angular gyrus	L	−42	−58	40	3.46
Anterior cingulate and paracingulate gyri	L	−2	42	6	3.14
	R	6	36	8	2.85
Superior frontal gyrus, medial	L	−8	62	8	2.96
Inferior parietal gyrus	L	−50	−58	46	2.81
Supramarginal gyrus	L	−56	−52	32	2.69

Coordinates are in MNI152 mm space. Results were thresholded at *Z* > 1.96 (*p* < 0.05) and corrected for multiple comparisons at the cluster level (*p* < 0.05)

*L* left, *R* right, *BIO* biological motion, *ODD* oppositional defiant disorder

### Neural correlates of disruptive behavior and autism symptom severity within ASD

**Fig. 4:**
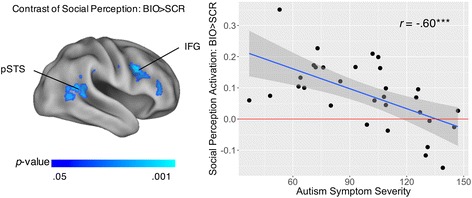
Neural correlates of autism symptom severity on the contrast of social perception (BIO > SCR) in ASD. Autism symptom severity was based on Social Responsiveness Scale (SRS) total raw scores, while controlling for oppositional defiant disorder (ODD) scores. *Left panel* illustrates the brain regions showing significant correlates. *Right panel* is the scatterplot of autism symptom severity (*x*-axis) and the average activations to BIO > SCR in these social perception brain regions (*y*-axis; unit: percent signal change), with a regression line and the 95% confidence intervals. BIO, biological motion; SCR, scrambled motion; pSTS, posterior superior temporal sulcus; IFG, inferior frontal gyrus. ***p < 0.001

The first analysis was on the contrast of social perception (BIO > SCR). As in the between-group analyses, we masked the analysis by a combined, inclusive (TD∪ASD) mask consisting of regions that showed main effects for BIO > SCR within either group (see Additional file 1). First, when ODD total scores and SRS total raw scores were examined separately, the analysis did not reveal any regions showing either positive or negative correlations between ODD total scores and the contrast of BIO > SCR, and there were no regions showing positive correlations with SRS total raw scores on this contrast, either. However, we found reliable negative correlations between SRS total raw scores and the contrast of BIO > SCR in the right IFG (694 voxels; see Additional file 3). Second, when ODD total scores and SRS total raw scores were examined simultaneously, there were also no regions showing either positive or negative correlations between ODD total scores and the contrast of BIO > SCR, and there were also no regions showing positive correlations with SRS total raw scores on this contrast. However, there were reliable negative correlations between SRS total raw scores and the contrast of BIO > SCR in the right pSTS and IFG (1426 voxels; Fig. 4; Table 5), such that children and adolescents with more severe autism symptoms showed less neural activations in these social perception regions. Importantly, the regions showing negative correlations between SRS total raw scores and the contrast of BIO > SCR—whether SRS total raw scores and ODD total scores were examined separately or simultaneously—were the same regions where social perception activation has been shown to be weaker in ASD (vs. TD) children [2–4]. In brief, consistent with the between-group results, the within-ASD results on the contrast of BIO > SCR showed that as autism symptom severity increases, activation decreases in key social perception regions, whereas there was no evidence that disruptive behavior is associated with social perception activation.

**Table 5:** Peaks of regions where the contrast of social perception (BIO > SCR) was negatively correlated with autism symptom severity

Anatomical regions		*x*	*y*	*z*	*Z*
Inferior frontal gyrus, opercular part	R	40	8	32	4.38
Inferior frontal gyrus, triangular part	R	36	20	24	4.51
Middle frontal gyrus	R	36	20	22	3.73
Precentral gyrus	R	42	6	32	3.80
Supramarginal gyrus	R	50	−40	30	3.51
Middle temporal gyrus	R	60	−60	6	3.31
Superior temporal gyrus	R	52	−48	20	3.43

Autism symptom severity was based on Social Responsiveness Scale (SRS) total raw scores while controlling for oppositional defiant disorder (ODD) scores. Coordinates are in MNI152 mm space. Results were thresholded at *Z* > 1.96 (*p* < 0.05) and corrected for multiple comparisons at the cluster level (*p* < 0.05)

*R* right, *BIO* biological motion, *SCR* scrambled motion

**Fig. 5:**
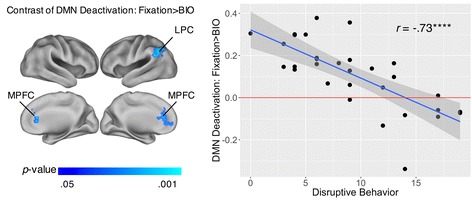
Neural correlates of disruptive behavior on the contrast of DMN deactivation (fixation > BIO) in ASD. Disruptive behavior was based on oppositional defiant disorder (ODD) scores, without controlling for Social Responsiveness Scale (SRS) total raw scores. *Left panel* illustrates the brain regions showing significant correlates. *Right panel* is the scatterplot of disruptive behavior (*x*-axis) and the average DMN deactivations to fixation > BIO in these brain regions (*y*-axis; unit: percent signal change), with a regression line and the 95% confidence intervals. DMN, default mode network; BIO, biological motion; MPFC, medial prefrontal cortex; LPC, lateral parietal cortex. ****p < 0.0001

The second analysis was based on the contrast of DMN deactivation (fixation > BIO). As in the between-group analyses, we masked the analysis by a combined, inclusive (TD∪ASD) mask consisting of regions that showed main effects for fixation > BIO within the DMN within either group (see Additional file 1). First, when ODD total scores and SRS total raw scores were examined separately, the analysis did not reveal any regions showing either positive or negative correlations between SRS total raw scores and the contrast of fixation > BIO, and there were no regions showing positive correlations with ODD total scores on this contrast, either. However, we found reliable negative correlations between ODD total scores and the contrast of fixation > BIO in the medial prefrontal cortex (MPFC) and lateral parietal cortex (LPC) (1277 voxels; Fig. 5; Table 6), such that children with more disruptive behavior showed less DMN deactivation in these regions. Second, when ODD total scores and SRS total raw scores were examined simultaneously, there were also no regions showing either positive or negative correlations between SRS total raw scores and the contrast of fixation > BIO, and there were also no regions showing positive correlations with ODD total scores on this contrast. However, there were reliable negative correlations between ODD total scores and the contrast of fixation > BIO in the left LPC (700 voxels; see Additional file 4). Notably, the regions showing negative correlations between ODD total scores and the contrast of fixation > BIO—whether SRS total raw scores and ODD total scores were examined separately or simultaneously—are completely non-overlapped with the regions that showed negative correlations between the contrast of BIO > SCR and SRS total raw scores. In brief, consistent with the between-group results, the within-ASD results on the contrast of DMN deactivation (fixation > BIO) showed that as disruptive behavior increases, DMN deactivation decreases in specific regions, whereas there is no evidence that autism symptom severity is associated with DMN deactivation.

**Table 6:** Peaks of regions in which the contrast of DMN deactivation (fixation > BIO) was negatively correlated with disruptive behavior within ASD

Anatomical regions		*x*	*y*	*z*	*Z*
Angular gyrus	L	−42	−58	40	3.27
Anterior cingulate and paracingulate gyri	L	0	40	26	3.04
	R	4	42	26	2.81
Superior frontal gyrus, medial orbital	L	−10	56	−2	2.52
Superior frontal gyrus, medial	L	0	42	26	3.01
Inferior parietal gyrus	L	−52	−58	48	3.46
Supramarginal gyrus	L	−60	−50	34	3.24

Disruptive behavior was based on oppositional defiant disorder (ODD) scores, without controlling for Social Responsiveness Scale (SRS) total raw scores. Coordinates are in MNI152 mm space. Results were thresholded at *Z* > 1.96 (*p* < 0.05) and corrected for multiple comparisons at the cluster level (*p* < 0.05)

*L* left, *R* Right, *BIO* biological motion, *DMN* default mode network

Based on the results from the second analysis showing the link between disruptive behavior and less deactivation in specific DMN regions in ASD, we further explored possible underlying mechanisms that might help explain this link. Specifically, we tested whether anxiety and ADHD symptoms, respectively, might mediate this link.

**Fig. 6:**
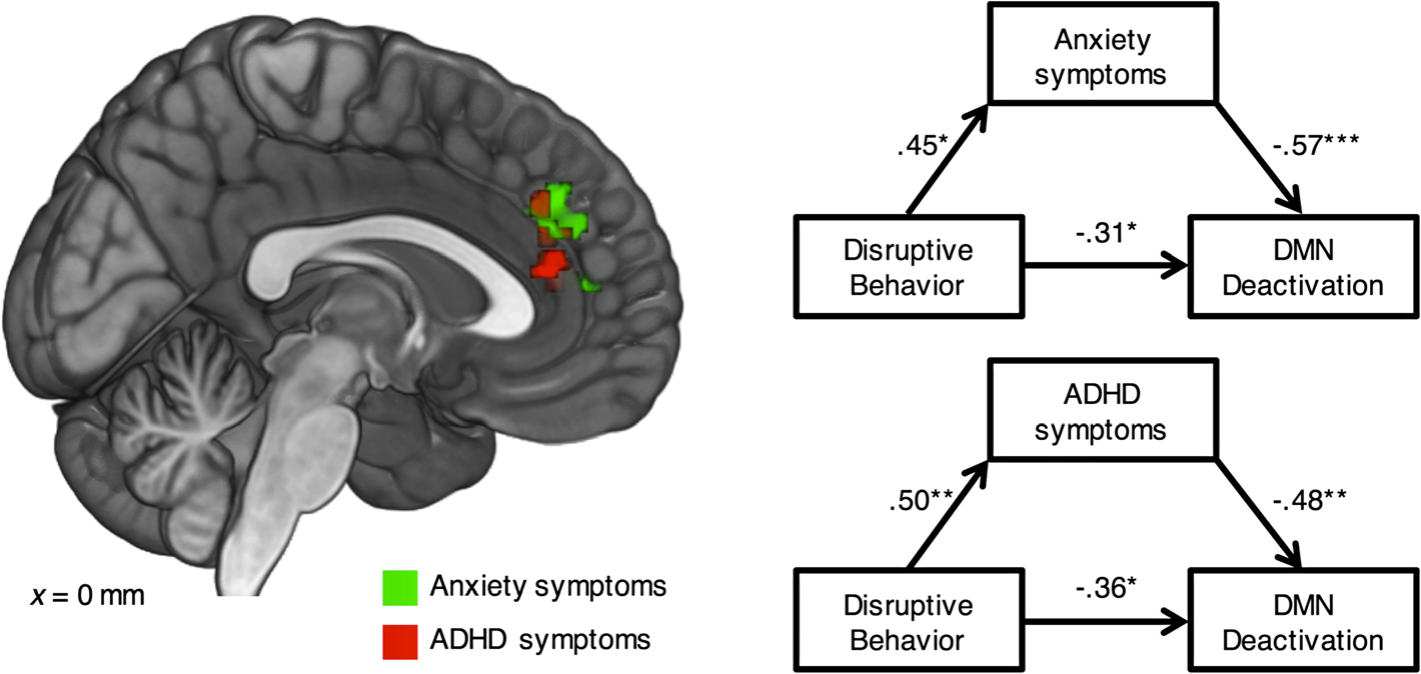
Anxiety symptoms and ADHD symptoms, respectively, mediated the link between disruptive behavior and less DMN deactivation in ASD. *Left panel* illustrates the brain regions showing the mediation effects. *Right panel* illustrates the corresponding mediation models. DMN, default mode network. *p < 0.05 **p < 0.01 ***p < 0.001

For anxiety symptoms, first, there was a significant correlation between disruptive behavior and anxiety symptoms, *r* = 0.45, *t*(29) = 2.72, *p* = 0.01, supporting the potency of anxiety symptoms as a mediator. Second, when we entered disruptive behavior and anxiety symptoms simultaneously as independent variables in GLM equation (6) on the contrast of DMN deactivation (fixation > BIO), and masked the analysis by the specific regions that showed the link between disruptive behavior and less DMN deactivation, the analysis revealed that anxiety symptoms partially mediated the link within the DMN in a cluster (188 voxels, 1504 mm^3^) primarily localized within the anterior cingulate cortex (ACC) and the medial part of the superior frontal gyrus (Fig. 6; Table 7), the completely standardized indirect effect = −0.26, 95% CI = [−0.43, −0.11], Sobel’s *Z* = −2.24, *p* = 0.03.

**Table 7:** Peaks of regions where ADHD and anxiety symptoms mediated the link between disruptive behavior and less DMN deactivation in ASD

Mediator	Anatomical regions		*x*	*y*	*z*	*Z*
Anxiety symptoms	Anterior cingulate and paracingulate gyri	L	−10	38	10	3.11
		R	6	38	28	2.67
	Middle cingulate and paracingulate gyri	R	2	36	30	2.63
	Superior frontal gyrus, medial	L	−2	38	28	2.69
		R	4	42	32	2.63
ADHD symptoms	Anterior cingulate and paracingulate gyri	L	2	38	14	3.16
		R	6	38	28	2.39
	Middle cingulate and paracingulate gyri	R	2	36	30	2.76
	Superior frontal gyrus, medial	L	2	34	32	2.78

Coordinates are in MNI152 mm space. Results were thresholded at *Z* > 1.96 (*p* < 0.05) and corrected for multiple comparisons at the cluster level (*p* < 0.05)

*L* left, *R* right, *DMN* default mode network

Finally, for ADHD symptoms, first, there was a significant correlation between disruptive behavior and ADHD symptoms, *r* = 0.50, *t*(29) = 3.11, *p* < 0.01, supporting the potency of ADHD symptoms as a mediator. Second, when we entered disruptive behavior and ADHD symptoms simultaneously as independent variables in GLM equation (7) on the contrast of DMN deactivation (fixation > BIO), and masked the analysis by the specific regions that showed the link between disruptive behavior and less DMN deactivation, the analysis revealed that ADHD symptoms partially mediated the link within the DMN in a cluster (57 voxels, 456 mm^3^) primarily localized in ACC (Fig. 6; Table 7), the completely standardized indirect effect = −0.24, 95% CI = [−0.55, −0.07], Sobel’s *Z* = −2.17, *p* = 0.03. There was a small overlapped region (25 voxels, 200 mm^3^) between the mediating regions of anxiety symptoms (13.30%) and those of ADHD symptoms (43.86%). In brief, these results provide the evidence that both anxiety and ADHD symptoms partially and focally mediated the link between disruptive behavior and less DMN deactivation in ASD.

### Analyses on the contrast of fixation > SCR and that of SCR > BIO

While the contrast of fixation > BIO showed large regions of DMN deactivation and was useful in revealing the neural basis of disruptive behavior in ASD, it remained unclear to what extent the results were specific to this contrast. To address this issue, we conducted follow-up analyses using GLM equations (1) and (4) on the contrast of fixation > SCR and that of SCR > BIO, respectively. First, on the contrast of fixation > SCR, the results showed that there were no DMN regions in the TD group and relatively limited DMN regions in the ASD group (see Additional file 5), while there were no DMN regions showing the link between ODD total scores and less DMN deactivation in ASD on this contrast. Second, on the contrast of SCR > BIO, the results showed that there were also large DMN regions in both TD and ASD groups and the regions were similar to the findings with fixation > BIO, while there were also negative correlations between ODD total scores and less DMN deactivation in ASD in the MPFC and left LPC regions (see Additional file 5).

## Discussion

Disruptive behavior in children with ASD is an important clinical problem, and the symptoms often impact overall functioning and exacerbate psychosocial impairment [14]. Better defining the neural basis of disruptive behavior in ASD and its relationship with the core symptoms of ASD may help identify targets for more effective treatment (e.g., improved and more specific behavioral and pharmacological interventions). To our knowledge, the current study is the first to examine the neural underpinning of disruptive behavior in ASD.

In terms of neural correlates, our results first showed that as expected, the ASD group (vs. TD)—whether it was the overall ASD sample, or ASD subgroups of high or low disruptive behavior—consistently showed hypoactivation in well-established social information processing regions such as the right pSTS and FFG, while providing no evidence that hypoactivation in ASD in these regions changes as a function of disruptive behavior. This provides extended support that hypoactivation in the social perception circuitry is tied with core ASD symptoms [2–4] and provides preliminary evidence that the hypoactivation can be observed across high or low levels of disruptive behavior.

Furthermore, as expected, the contrast of fixation > BIO revealed large regions of DMN deactivation in both TD and ASD groups. Within these regions, we found that ASD with high disruptive behavior (vs. ASD with low disruptive behavior or TD) showed more restricted regions and less DMN deactivation, suggesting that DMN deactivation in ASD changes as a function of disruptive behavior and that there is DMN abnormality in the ASD subgroup with high disruptive behavior. In addition, consistent with the between-group findings, the within-ASD dimensional analyses showed that whereas autism symptom severity (but not disruptive behavior) was uniquely associated with less social perception activation in the right pSTS and IFG, disruptive behavior (but not autism symptom severity) was uniquely associated with less DMN deactivation in the MPFC and LPC. In brief, the results provide the doubly dissociable evidence that disruptive behavior and autism symptom severity in children and adolescents with ASD have distinct, separable neural bases. Critically, these findings imply that differential treatment should be provided to treat disruptive behavior in ASD and the treatment could aim at improving DMN functions (e.g., [72]), while DMN deactivation may also be tested as a neural predictor or mechanism of behavioral response to treatment [73].

While the contrast of fixation > BIO is useful in revealing the neural basis of disruptive behavior in ASD, follow-up analyses on the contrasts of fixation > SCR and SCR > BIO provide additional insights into its generality. First, on the contrast of fixation > SCR, there were relatively limited DMN deactivations in the ASD group, and no regions of DMN deactivations in the TD group. This suggests that SCR (vs. fixation) may not be as critically cognitively demanding and requiring active suppression of self-referential thoughts as BIO (vs. fixation) and may be ill-suited to test the neural basis of disruptive behavior in ASD. Second, on the contrast of SCR > BIO, there were also large regions of DMN deactivation in either TD or ASD group, and within the ASD group, disruptive behavior was negatively correlated with DMN deactivation in MPFC and LPC. This suggests that the contrast of SCR > BIO is also useful in detecting DMN deactivation and revealing the neural basis of disruptive behavior in ASD. However, we argue that the contrast of fixation > BIO affords a more straightforward interpretation of DMN deactivation than SCR > BIO because fixation (compared to SCR) involves relatively minimal external stimuli.

In the within-ASD dimensional analyses, it is intriguing that SRS total raw scores and ODD total scores were found to be marginally correlated in ASD. While this suggests some discriminant validity of these two entities and that disruptive behavior is a relatively distinct comorbidity of ASD, rather than just a manifestation of the core symptoms of ASD, the finding also suggests that there was some overlap in the two measures. This is consistent with the past findings that SRS-parent total scores were higher in clinical populations with ADHD and/or conduct disorders (CD) [60] and SRS-parent total scores were better at differentiating ASD and TD than differentiating ASD and ODD/CD [74]. One possible explanation is that the SRS-parent total scores may measure social impairments in general, rather than exclusively ASD symptomatology, and it is likely that a child or adolescent with high ODD would also have affected social skills and thus elevated SRS total scores. Accordingly, a purer measure of autism symptom severity might be SRS total raw scores partialling out ODD total scores. Although speculative, this possibility is consistent with our finding that before we controlled for ODD total scores, SRS total raw scores were found to relate to less social perception activation in only the right IFG; however, after controlling for ODD total scores, less social perception activation was found in the right IFG as well as the right pSTS. By the same logic, partialling out SRS total raw scores would potentially remove some variance of disruptive behavior in the ODD total scores. Again, although speculative, this possibility is consistent with our finding that before we controlled for SRS total raw scores, ODD total scores were found to relate to less DMN deactivation in the LPC and MPFC; however, after controlling for SRS total raw scores, less DMN deactivation was found only in the LPC.

While it remains a matter of debate whether disruptive behavior in children with ASD is an epiphenomenon (i.e., pleiotropic manifestations of the ASD diathesis), phenocopy (i.e., induced by living in an environment due to having ASD symptoms), or co-morbid psychiatry entity that is distinct from ASD itself, previous literature shows that the psychopathology of children with disruptive behavior is similar between ASD and non-ASD control samples [63], which suggests that the etiology of disruptive behavior in ASD may be separable from that of the core symptoms of ASD. Our results are consistent with this previous behavioral finding and further provide the first neural evidence that the endophenotype of disruptive behavior in ASD is differentiable from that of core ASD symptoms in ASD.

Our results also showed that anxiety and ADHD symptoms, respectively, mediate the link between disruptive behavior and less DMN deactivation in ASD in the MPFC and ACC, with ADHD symptoms playing more of a role in the ACC, and anxiety symptoms in both ACC and MPFC. The ACC has been implicated in stimulus selection (focusing attention) and response selection (related to inhibiting impulsivity) and dysfunctional in ADHD [75]. The MPFC has been implicated in self-referential processing and self-esteem or positive/negative self-endorsement and dysfunctional in anxiety disorder [76, 77]. Our findings thus help explain the possible neural mechanisms of how disruptive behavior in ASD is related to less DMN deactivation, and may provide targets for even more precise interventions in ASD children with disruptive behavior. It should be noted that the evidence for the mediating effects is limited to MPFC and ACC but not other DMN regions such as LPC. Indeed, more research is needed to understand the full neural mechanisms underlying disruptive behavior in ASD.

Currently, the Food and Drug Administration (FDA) has approved two atypical antipsychotic drugs for treating irritability and disruptive behavior associated with autism: risperidone [78, 79] and aripiprazole [80, 81]. However, neither drug has been shown to be effective in improving the core ASD symptoms, particularly social communicative impairments [82]. Our results suggest that disruptive behavior and core ASD symptoms have distinct and separable neural processes, which leads to the hypothesis that the neural mechanisms of these two drugs are specifically related to the DMN but not the social perceptual processes; future research can test this hypothesis. Furthermore, our results provide the evidence that social perceptual neural processes should be the target for treating core ASD symptoms. Recently, for example, oxytocin was found to improve brain function in children with ASD [83] in the same brain regions identified as underlying social perception in the current study (pSTS and IFG). In sum, the neural bases revealed in this research may serve as differential neurobiological markers when developing or evaluating behavioral and pharmacological interventions in ASD.

As it is characterized by frequent angry outburst and irritability, disruptive behavior may be more broadly related to mood dysregulation [84] as well as poor self-regulation [85], including deficits in self-monitoring, self-control, and self-management [86–88]. While our biological motion task provides a window into DMN deactivation, it awaits to be tested how disruptive behavior in ASD may be related to other self-regulatory neural systems, such as the meta-cognitive system [89], the orbitofrontal-amygdala circuit [90], the executive functioning circuit [91]. Similarly, while our task may be more cognitive, an important future direction is to use a task that may more actively induce frustration and requires mood regulation (e.g., a Go/No-Go task with high difficulty [92]), which may require proper functioning of the paralimbic system that regulates motivation and affect [93]. Future works may consider these directions.

### Limitations

Several limitations are important to consider in this research. First, recently, it has been shown that there may be higher false positive rates when the fMRI parametric analyses are based on a weaker CDT in single studies, except for FSL’s FLAME1 [94]. Our confidence in the current results is boosted because we used FSL’s FLAME1 + 2 to estimate the results, the peak voxel-level significance in our results is mostly very high, *Z* > 3.09, *p* < 0.001, the effects were hypothesized rather than completely data-driven, and importantly, the results replicate the past findings that ASD is associated with social perception deficits [2–4] and disruptive behavior is associated with DMN abnormality [42–45], while replication is a widely accepted method for establishing true effects [68]. Nonetheless, future works should use a more stringent CDT in order to reduce the concern of Type I error, which will also require a larger sample as well in order to reduce Type II error [95]. Second, out of the 31 participants with ASD, only 7 (23%) were high ODD. The sample size of this critical subgroup is relatively small and future work should include a larger ASD sample with high ODD to further establish the between-group findings. Nevertheless, this subgroup was based on clinically meaningful cutoffs, the percentage is consistent with the prevalence of disruptive behavior disorder in children with ASD [10], and the between-group analysis involving the subgroup was not standalone but further supported by the within-ASD dimensional analysis. Third, about 35% of the participants also participated in a prior imaging study [3] that investigated the neural basis of ASD. Future research should recruit completely independent samples to further test the neural basis of ASD, although the majority (65%) of the data did not overlap with that study and the current research provided a preliminary, yet incremental understanding that hypoactivation in the key social perception regions can be observed in ASD subgroups with high or low levels of disruptive behavior.

Fourth, the current design was non-factorial and there were no participants of high ODD without ASD; future work can test whether the observed effect of disruptive behavior on less DMN deactivation also holds in a non-ASD population. Fifth, all ratings of clinical symptoms were made by parental reports (SRS, ODD, anxiety, ADHD), which is a limitation of the methodology; only one perspective of the child was obtained, and it is possible that parents may not fully appreciate the subtle differences in symptomatology between ASD and other common comorbidities, such as reduced attention as a function of ASD, not necessarily a pure deficit in attention. Future works should include both parental and clinician ratings. Sixth, we used the biological motion task to tap into DMN deactivation. However, the fixation periods were relatively short and were at special locations (20 s at beginning and 16 s at the end), and the BIO may be only moderately cognitively demanding. Furthermore, it is primarily about DMN deactivation and it remains unclear how the results may generalize to DMN connectivity. Further research is needed to increase the length of fixation periods, for example, by adding jittered fixation periods between BIO and SCR blocks, use a more cognitively demanding task, and should also test whether the link between disruptive behavior and DMN atypicality in ASD can be observed in other fMRI tasks (e.g., [92]) or resting-state connectivity analyses (e.g., [96–99]).

Seventh, the current study included a sample of high functioning individuals with ASD (IQ > 70). It is unclear whether the findings could generalize to lower functioning individuals with ASD [100]. Eighth, our sample consists of children and adolescents 4–18 years of age, and future works may test whether the findings could generalize to adults with ASD [101]. Finally, all the participants with ASD were male. Prevalence of mood and anxiety disorders tends to be higher in girls with ASD relative to boys with ASD, especially during adolescence [102]. Future studies should expand the scope of participants to include females with ASD to inform gender-general or gender-specific neural correlates of disruptive behavior in ASD.

## Conclusions

Despite the limitations, the current study is the first to investigate the neural underpinnings of disruptive behavior in ASD, which could lead to the development of more precise medicine in ASD. Our results suggest that while core ASD symptoms are related to hypoactivation in the social perception circuitry, disruptive behavior in ASD has a distinct neural basis that is separable from core ASD symptoms and characterized by less deactivation in the DMN. Accordingly, differential treatments should be provided to treat disruptive behavior in ASD. For example, increasing DMN deactivation might be a possible direction for developing novel neuroscience-based interventions for disruptive behavior in ASD. Furthermore, DMN deactivation may be used as a biomarker to evaluate or predict the effectiveness of behavioral and pharmacological treatments for disruptive behavior in ASD.

## Additional files

Additional file 1: Inclusive mask of the main effects on the contrast of social perception (BIO > SCR) and that of DMN deactivation (fixation > BIO). Inclusive (union) mask of the main effects within either TD or ASD group: The contrast of social perception (BIO > SCR) and that of DMN deactivation (fixation > BIO). (PDF 1314 kb)
Additional file 2: Brain regions showing DMN deactivations to fixation > BIO in TD and ASD, respectively. Table showing the peaks of regions of deactivation during biological motion vs. during fixation (fixation > BIO) within the default mode network (DMN) in TD and ASD, respectively. (PDF 77 kb)
Additional file 3: Neural correlates of Social Responsiveness Scale (SRS) total raw scores without controlling for oppositional defiant disorder (ODD) total scores on the contrast of social perception (BIO > SCR) in ASD. Table and figure showing the negative correlation between SRS total raw scores without controlling for ODD total scores and the average social perception activations to BIO > SCR in ASD. (PDF 539 kb)
Additional file 4: Neural correlates of oppositional defiant disorder (ODD) total scores, controlling for Social Responsiveness Scale (SRS) total raw scores, on the contrast of DMN deactivation (fixation > BIO) in ASD. Table and figure showing the negative correlation between ODD total scores, controlling for SRS total raw scores, and the average DMN deactivations to fixation > BIO in ASD. (PDF 523 kb)
Additional file 5: Results on the contrast of fixation > SCR and that of SCR > BIO. (1) Group main effects of the contrast of fixation > SCR within DMN; (2) group main effects of the contrast of SCR > BIO within DMN; (3) neural correlates of oppositional defiant disorder (ODD) total scores without controlling for Social Responsiveness Scale (SRS) total raw scores on the contrast of SCR > BIO within DMN in ASD. (PDF 1599 kb)

## Abbreviations

ACC: Anterior cingulate cortex
ADHD: Attention-deficit/hyperactivity disorder
ADI-R: Autism Diagnostic Interview-Revised
ADOS: Autism Diagnostic Observation Schedule
ASD: Autism spectrum disorder
BIO: Biological motion
CDT: Cluster-defining threshold
DMN: Default mode network
FFG: Fusiform gyrus
GLM: General linear model
IFG: Inferior frontal gyrus
IQ: Intelligence quotient
LPC: Lateral parietal cortex
MPFC: Medial prefrontal cortex
ODD: Oppositional defiant disorder
pSTS: Posterior superior temporal sulcus
SCR: Scrambled motion
SRS: Social Responsiveness Scale
TD: Typically developing
