# Supplementary material for: Distinct neural bases of disruptive behavior and autism symptom severity in boys with autism spectrum disorder
Source: J Neurodev Disord. 2017 Jan 17;9:1. doi: 10.1186/s11689-017-9183-z (PMC5240249; doi:10.1186/s11689-017-9183-z)

Additional file 1

Inclusive mask of the main effects within either TD or ASD group: The contrast of social perception (BIO>SCR)

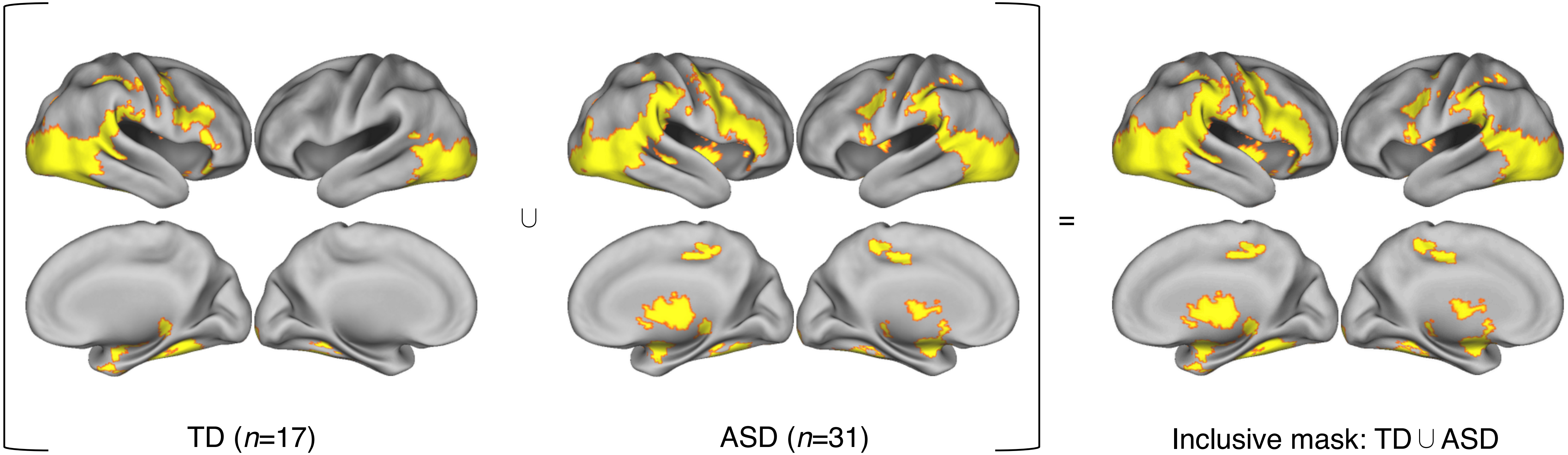

Inclusive mask of the main effects within either TD or ASD group: The contrast of DMN deactivation (Fixation>BIO)

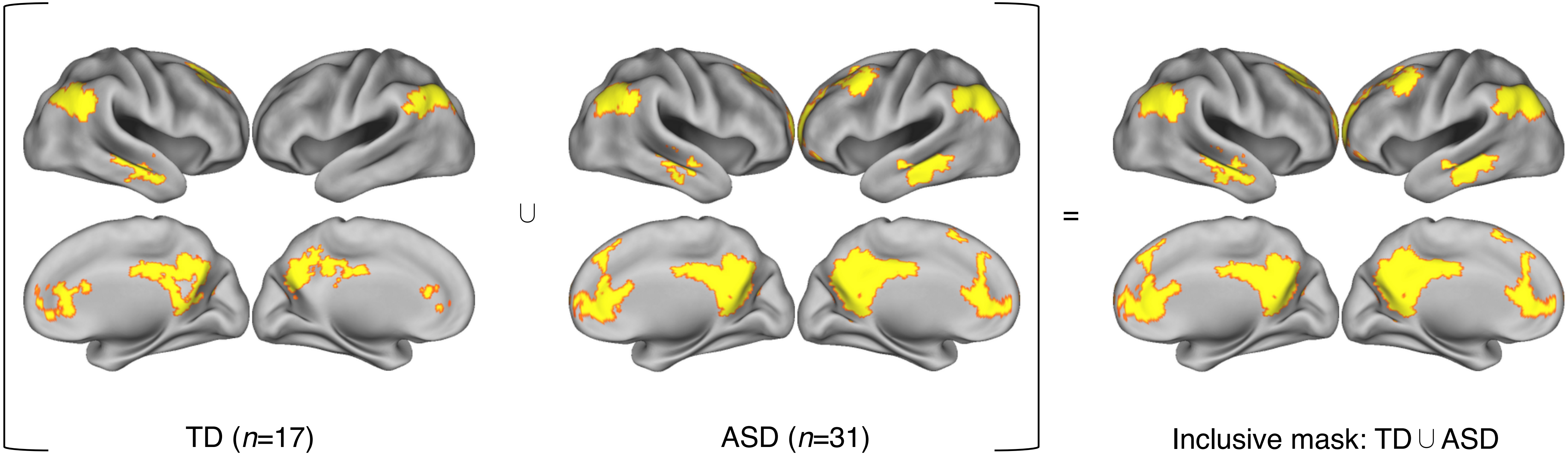

Supplement: Additional file 1: — Inclusive mask of the main effects on the contrast of social perception (BIO > SCR) and that of DMN deactivation (fixation > BIO). Inclusive (union) mask of the main effects within either TD or ASD group: The contrast of social perception (BIO > SCR) and that of DMN deactivation (fixation > BIO). (PDF 1314 kb) [file 11689_2017_9183_MOESM1_ESM.pdf]
