# Supplementary material for: Distinct neural bases of disruptive behavior and autism symptom severity in boys with autism spectrum disorder
Source: J Neurodev Disord. 2017 Jan 17;9:1. doi: 10.1186/s11689-017-9183-z (PMC5240249; doi:10.1186/s11689-017-9183-z)
Supplement: Additional file 2: — Brain regions showing DMN deactivations to fixation > BIO in TD and ASD, respectively. Table showing the peaks of regions of deactivation during biological motion vs. during fixation (fixation > BIO) within the default mode network (DMN) in TD and ASD, respectively. (PDF 77 kb) [file 11689_2017_9183_MOESM2_ESM.pdf]

Additional file 2

Table: Peaks of regions of deactivation during biological motion versus during fixation (fixation>BIO) within the Default Mode Network (DMN) in TD and ASD, respectively

Anatomical regions		TD				ASD			
		x	*y*	*z*	*Z*	*x*	*y*	*z*	*Z*
Angular gyrus	L	-42	-70	42	3.84	-40	-68	46	5.81
	R	58	-52	30	4.14	48	-60	48	5.52
Calcarine fissure and surrounding cortex	L	0	-68	20	3.08	-12	-62	10	6.09
	R	6	-60	18	2.65	6	-62	16	6.61
Anterior cingulate & paracingulate gyri	L	2	40	18	3.34	2	46	6	4.32
	R	2	38	18	3.53	2	44	6	4.56
Middle cingulate & paracingulate gyri	L	0	-44	46	4.04	0	-44	46	5.32
	R	2	-44	36	3.96	2	-44	36	4.76
Posterior cingulate gyrus	L	0	-40	32	2.63	0	-34	28	4.57
	R	8	-40	28	3.26	8	-42	22	4.66
Cuneus	L	2	-68	26	4.02	0	-74	30	6.68
	R	6	-70	26	4.41	6	-70	26	5.70
Superior frontal gyrus, medial orbital	L					-14	64	-2	3.02
	R	6	44	-6	2.67	4	46	-2	4.10
Middle frontal gyrus	L					-48	18	42	4.38
	R	26	36	36	3.54	32	30	50	4.23
Superior frontal gyrus, dorsolateral	L					-30	58	0	4.44
	R	24	44	38	4.66	28	32	50	4.19

Superior frontal gyrus, medial	L	2	44	22	2.99	0	30	32	4.22
	R	6	56	6	2.69	4	48	2	4.73
Lingual gyrus	L					-12	-54	2	5.30
	R	8	-42	4	2.98	12	-56	8	5.85
Lateral orbital gyrus	L					-42	48	-16	2.75
Middle occipital gyrus	L	-38	-80	38	3.34	-36	-70	40	4.43
	R					44	-72	32	3.25
Superior occipital gyrus	L	-14	-68	28	2.99	-14	-68	28	4.97
Inferior parietal gyrus	L	-44	-60	50	3.56	-36	-72	46	5.24
	R	58	-52	40	4.25	46	-58	44	4.58
Superior parietal gyrus	L	-38	-66	52	3.15	-36	-70	52	4.15
Precentral gyrus	L					-44	10	50	3.14
Precuneus	L	2	-74	38	4.21	-2	-70	42	6.74
	R	8	-72	36	4.88	4	-74	38	5.88
Supplementary motor area	L					0	12	60	3.27
Supramarginal gyrus	L	-58	-56	30	2.61	-60	-54	30	2.86
	R	52	-44	42	3.76	52	-44	44	3.33
Inferior temporal gyrus	L					-60	-26	-18	3.07
	R	66	-14	-26	3.03	64	-26	-18	3.01
Middle temporal gyrus	L					-64	-22	-14	3.47
	R	70	-14	-16	3.34	64	-10	-12	4.08
Superior temporal gyrus	R	68	-28	0	3.00	64	-12	-10	3.42

Note. Coordinates are in MNI152 mm space. Results were thresholded at $Z>1.96$ ($p<.05$) and corrected for multiple comparisons at the cluster level ($p<.05$). L, Left; R, Right.
