# Supplementary material for: Distinct neural bases of disruptive behavior and autism symptom severity in boys with autism spectrum disorder
Source: J Neurodev Disord. 2017 Jan 17;9:1. doi: 10.1186/s11689-017-9183-z (PMC5240249; doi:10.1186/s11689-017-9183-z)

Additional file 3

Table: Peaks of regions where the contrast of social perception (BIO>SCR) was negatively correlated with Social Responsiveness Scale (SRS) total raw scores in ASD, without controlling for Oppositional Defiant Disorder (ODD) total scores

Anatomical regions		*x*	*y*	*z*	*Z*
Inferior frontal gyrus, opercular part	R	38	8	32	4.38
Inferior frontal gyrus, triangular part	R	40	22	24	3.51
Middle frontal gyrus	R	38	8	36	3.69
Precentral gyrus	R	42	6	32	3.81

Note. Coordinates are in MNI152 mm space. Results were thresholded at $Z>1.96$ ($p<.05$) and corrected for multiple comparisons at the cluster level ($p<.05$). R, Right; BIO, Biological motion; SCR, Scrambled motion.

Figure: Neural correlates of autism symptom severity on the contrast of social perception (BIO>SCR) in ASD. Autism symptom severity was based on Social Responsiveness Scale (SRS) total raw scores, without controlling for Oppositional Defiant Disorder (ODD) total scores. Left panel illustrates the brain regions showing significant correlates. Right panel is the scatterplot of autism symptom severity (*x*-axis) and the average social perception activations to BIO>SCR in these brain regions (*y*-axis; unit: percent signal change), with a regression line and the 95% confidence intervals. BIO, Biological motion; SCR, Scrambled motion; IFG, inferior frontal gyrus. ****$p < .0001$

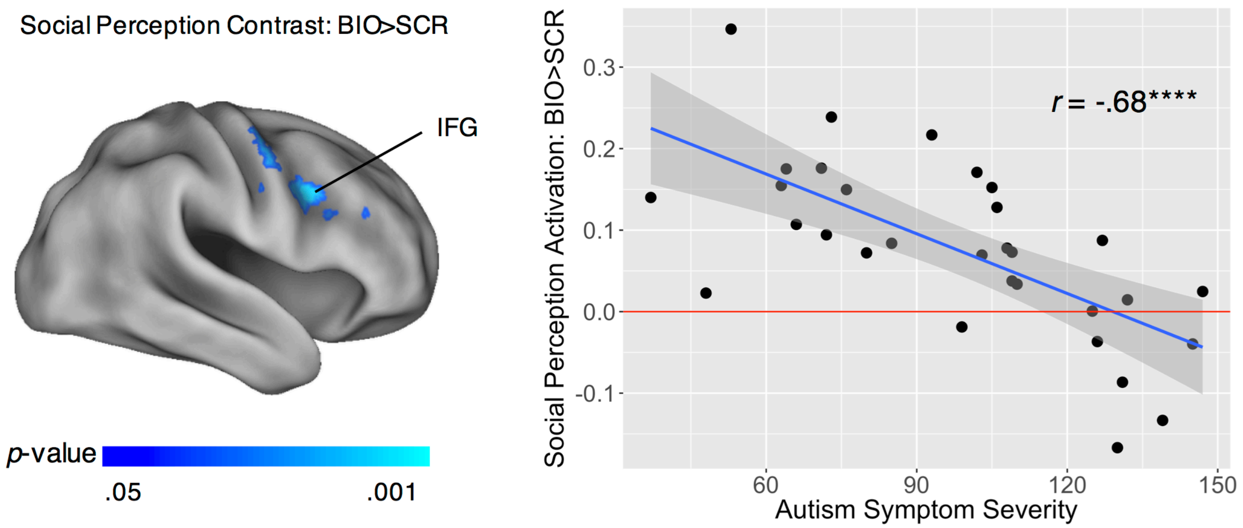

Supplement: Additional file 3: — Neural correlates of Social Responsiveness Scale (SRS) total raw scores without controlling for oppositional defiant disorder (ODD) total scores on the contrast of social perception (BIO > SCR) in ASD. Table and figure showing the negative correlation between SRS total raw scores without controlling for ODD total scores and the average social perception activations to BIO > SCR in ASD. (PDF 539 kb) [file 11689_2017_9183_MOESM3_ESM.pdf]
