# Supplementary material for: Distinct neural bases of disruptive behavior and autism symptom severity in boys with autism spectrum disorder
Source: J Neurodev Disord. 2017 Jan 17;9:1. doi: 10.1186/s11689-017-9183-z (PMC5240249; doi:10.1186/s11689-017-9183-z)

Additional file 4

Table: Peaks of regions in which the contrast of DMN deactivation (fixation>BIO) was negatively correlated with Oppositional Defiant Disorder (ODD) scores in ASD, while controlling for Social Responsiveness Scale (SRS) total raw scores

Anatomical regions		*x*	*y*	*z*	*Z*
Angular gyrus	L	-42	-58	40	3.14
Inferior parietal gyrus	L	-52	-58	48	3.45
Supramarginal gyrus	L	-56	-52	32	3.20

Note. Coordinates are in MNI152 mm space. Results were thresholded at $Z>1.96$ ($p<.05$) and corrected for multiple comparisons at the cluster level ($p<.05$). L, Left; BIO, Biological motion; DMN, Default Mode Network.

Figure: Neural correlates of disruptive behavior on the contrast of DMN deactivation (fixation>BIO) in ASD. Disruptive behavior was based on Oppositional Defiant Disorder (ODD) scores, while controlling for Social Responsiveness Scale (SRS) total raw scores. Left panel illustrates the brain regions showing significant correlates. Right panel is the scatterplot of disruptive behavior (*x*-axis) and the average DMN deactivations to fixation>BIO in these brain regions (*y*-axis; unit: percent signal change), with a regression line and the 95% confidence intervals. DMN, Default Mode Network; BIO, Biological motion; LPC, Lateral Parietal Cortex. $^{***}p < .001$

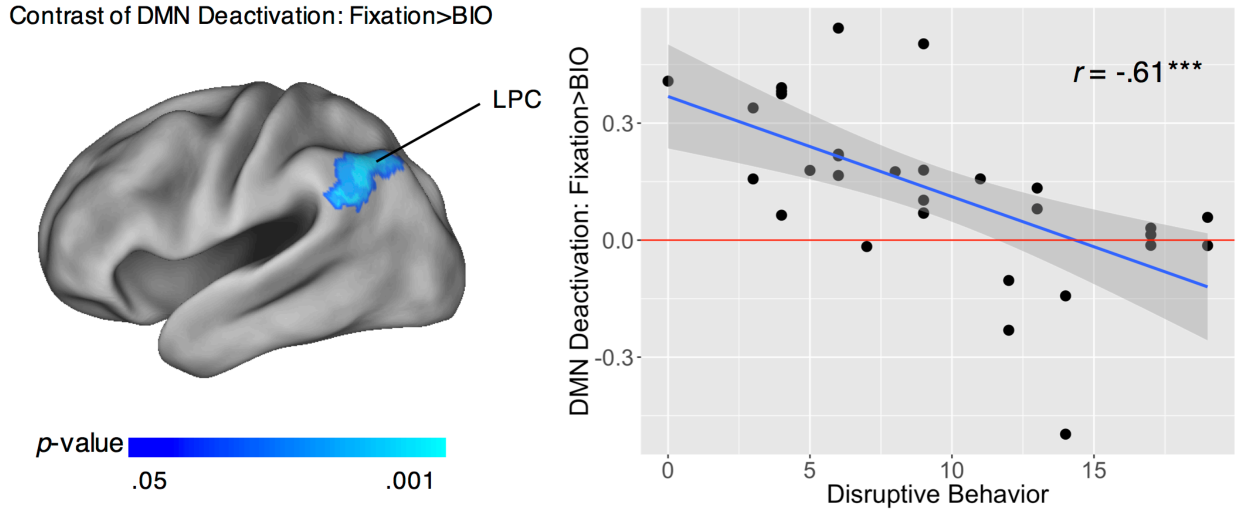

Supplement: Additional file 4: — Neural correlates of oppositional defiant disorder (ODD) total scores, controlling for Social Responsiveness Scale (SRS) total raw scores, on the contrast of DMN deactivation (fixation > BIO) in ASD. Table and figure showing the negative correlation between ODD total scores, controlling for SRS total raw scores, and the average DMN deactivations to fixation > BIO in ASD. (PDF 523 kb) [file 11689_2017_9183_MOESM4_ESM.pdf]
