# Supplementary material for: Distinct neural bases of disruptive behavior and autism symptom severity in boys with autism spectrum disorder
Source: J Neurodev Disord. 2017 Jan 17;9:1. doi: 10.1186/s11689-017-9183-z (PMC5240249; doi:10.1186/s11689-017-9183-z)

Additional file 5

Group main effects of the contrast of fixation>SCR within DMN

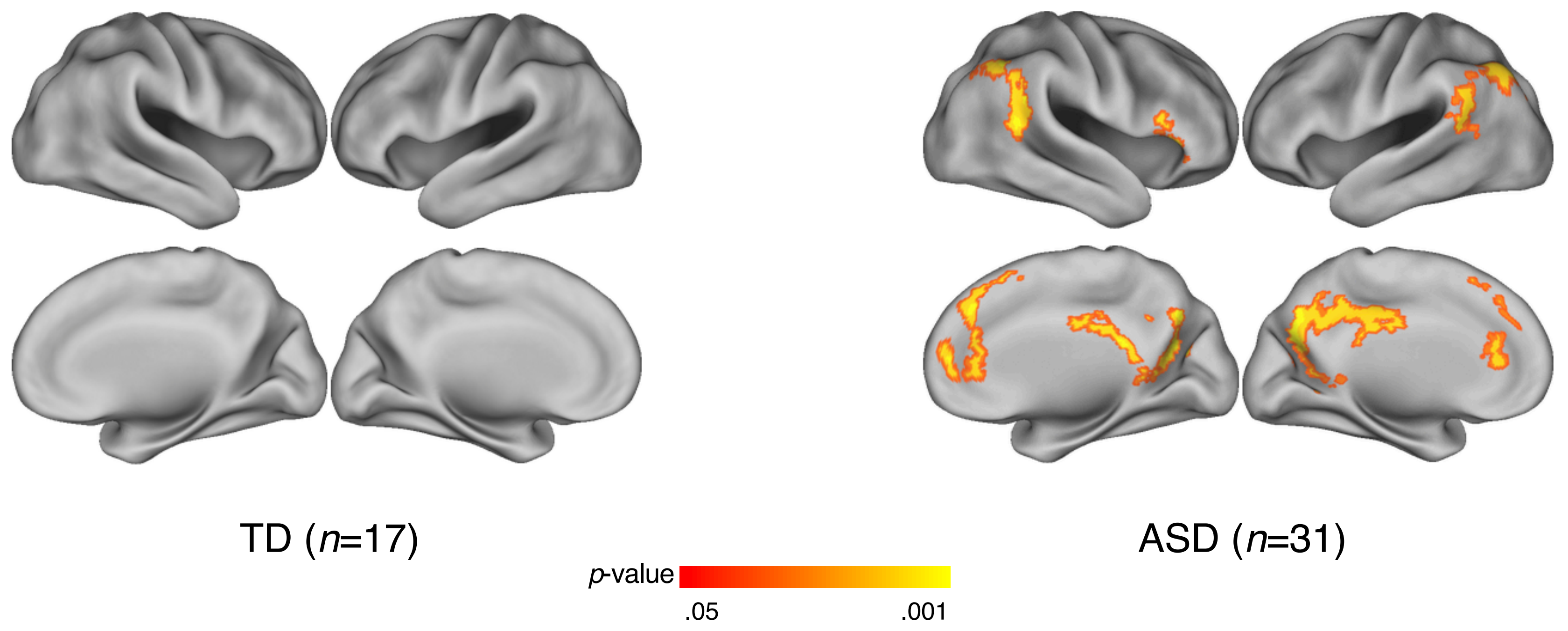

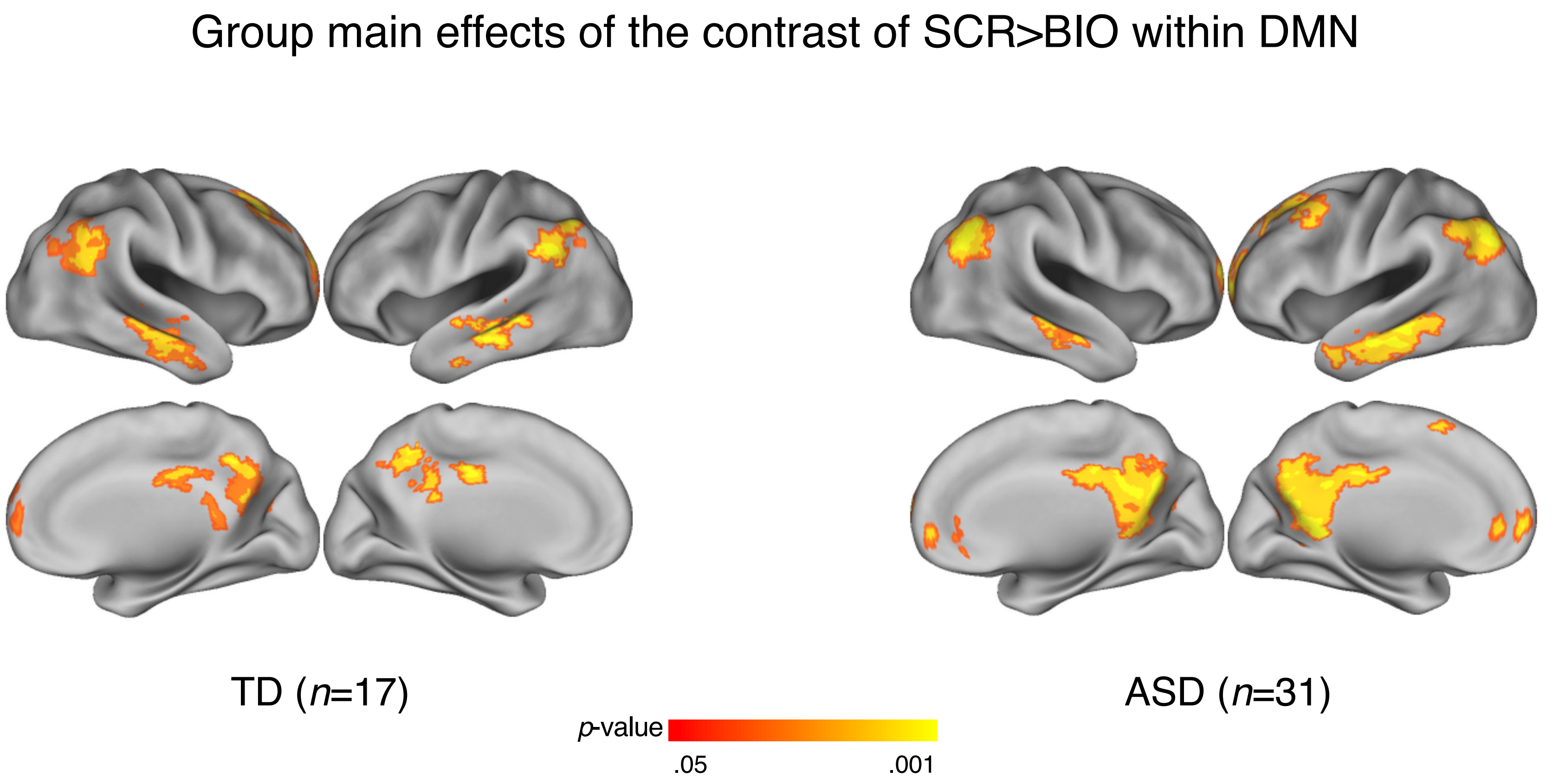
Group main effects of the contrast of SCR>BIO within DMN
TD (n=17)
ASD (n=31)
p-value
.05
.001

Neural correlates of disruptive behavior on the contrast of SCR>BIO within DMN in ASD

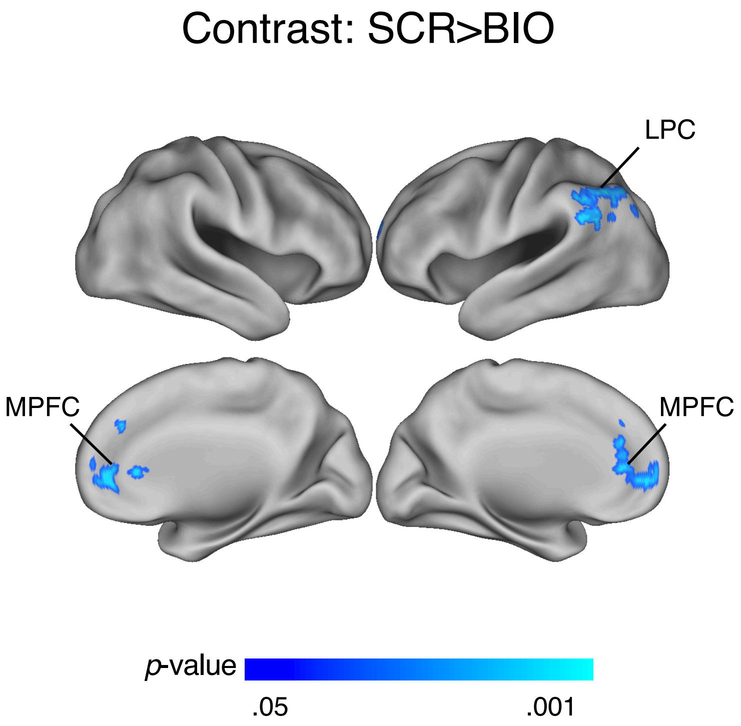

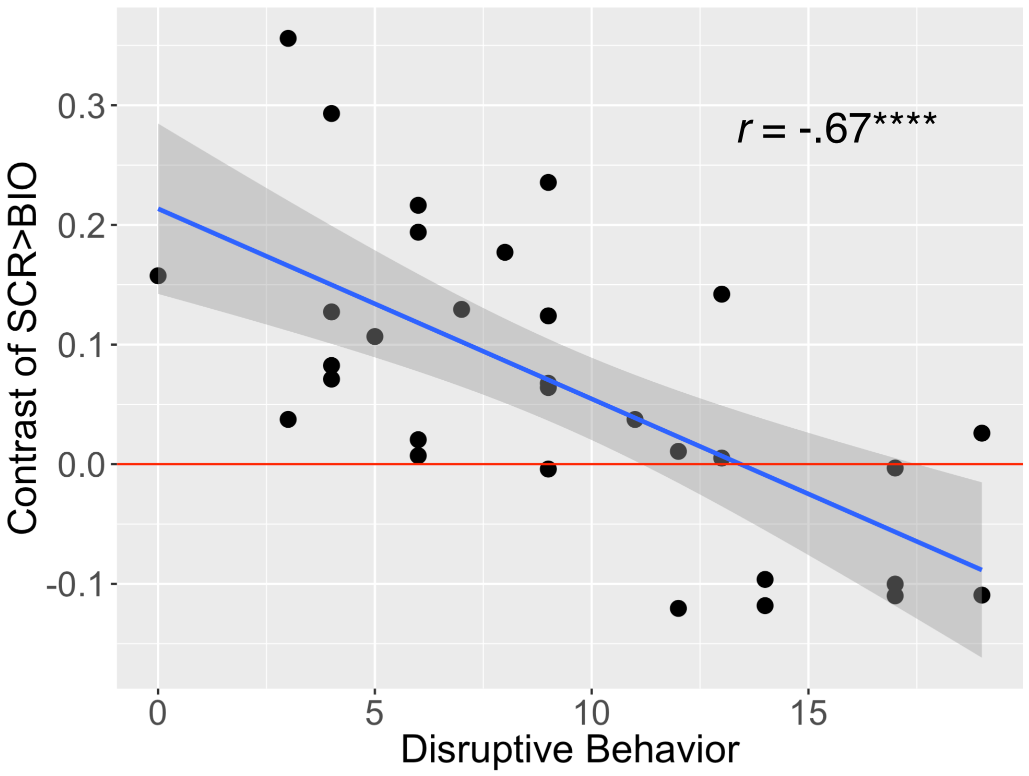

Supplement: Additional file 5: — Results on the contrast of fixation > SCR and that of SCR > BIO. (1) Group main effects of the contrast of fixation > SCR within DMN; (2) group main effects of the contrast of SCR > BIO within DMN; (3) neural correlates of oppositional defiant disorder (ODD) total scores without controlling for Social Responsiveness Scale (SRS) total raw scores on the contrast of SCR > BIO within DMN in ASD. (PDF 1599 kb) [file 11689_2017_9183_MOESM5_ESM.pdf]
